# Hypoxia Preconditioned Serum Hydrogel (HPS-H) Accelerates Dermal Regeneration in a Porcine Wound Model

**DOI:** 10.3390/gels10110748

**Published:** 2024-11-17

**Authors:** Jun Jiang, Tanita Man, Manuela Kirsch, Samuel Knoedler, Kirstin Andersen, Judith Reiser, Julia Werner, Benjamin Trautz, Xiaobin Cong, Selma Forster, Sarah Alageel, Ulf Dornseifer, Arndt F. Schilling, Hans-Günther Machens, Haydar Kükrek, Philipp Moog

**Affiliations:** 1 Experimental Plastic Surgery, Clinic for Plastic, Reconstructive and Hand Surgery, Klinikum Rechts der Isar, Technical University of Munich, 81675 Munich, Germany; 2 Center for Preclinical Research, Klinikum Rechts der Isar, TUM School of Medicine and Health, 81675 Munich, Germany; 3 Cellular Therapy and Immunobiology, Research and Innovation, King Faisal Specialist Hospital & Research Center, Al Mathar Ash Shamali, Riyadh 11564, Saudi Arabia; 4 Department of Plastic, Reconstructive and Aesthetic Surgery, Isar Klinikum, 80331 Munich, Germany; 5 Department of Trauma Surgery, Orthopedics and Plastic Surgery, University Medical Center Göttingen, 37075 Göttingen, Germany

**Keywords:** Hypoxia Preconditioned Serum, HPS, wound healing, angiogenesis, lymphangiogenesis, wound contraction, epithelialization, hyperspectral camera

## Abstract

Harnessing the body’s intrinsic resources for wound healing is becoming a rapidly advancing field in regenerative medicine research. This study investigates the effects of the topical application of a novel porcine Hypoxia Preconditioned Serum Hydrogel (HPS-H) on wound healing using a minipig model over a 21-day period. Porcine HPS exhibited up to 2.8× elevated levels of key angiogenic growth factors (VEGF-A, PDGF-BB, and bFGF) and demonstrated a superior angiogenic effect in a tube formation assay with human umbilical endothelial cells (HUVECs) in comparison to porcine normal serum (NS). Incorporating HPS into a hydrogel carrier matrix (HPS-H) facilitated the sustained release of growth factors for up to 5 days. In the in vivo experiment, wounds treated with HPS-H were compared to those treated with normal serum hydrogel (NS-H), hydrogel only (H), and no treatment (NT). At day 10 post-wounding, the HPS-H group was observed to promote up to 1.7× faster wound closure as a result of accelerated epithelialization and wound contraction. Hyperspectral imaging revealed up to 12.9% higher superficial tissue oxygenation and deep perfusion in HPS-H-treated wounds at day 10. The immunohistochemical staining of wound biopsies detected increased formation of blood vessels (CD31), lymphatic vessels (LYVE-1), and myofibroblasts (alpha-SMA) in the HPS-H group. These findings suggest that the topical application of HPS-H can significantly accelerate dermal wound healing in an autologous porcine model.

## 1. Introduction

Chronic wounds present a significant global healthcare challenge, affecting millions of individuals each year and contributing to substantial morbidity and healthcare costs [1]. These wounds, often arising from underlying conditions such as diabetes, vascular disease, or immune disorders, exhibit a complex and multifactorial pathophysiology [2]. Unlike acute wounds, chronic wounds present a hostile microenvironment characterized by persistent inflammation, elevated protease levels, extracellular matrix degradation, and impaired cellular function, that fails to progress through normal healing stages [3]. Despite advances in medical interventions, the management of chronic wounds remains difficult, highlighting the need for innovative treatment strategies [4].

Blood-derived growth factor therapy represents a promising approach in wound healing, utilizing the regenerative properties of natural factor compositions to accelerate tissue repair [5]. Key growth factors, such as platelet-derived growth factor BB (PDGF-BB), basic fibroblast growth factor (bFGF), and vascular endothelial growth factor (VEGF), play pivotal roles in driving cellular processes like proliferation and angiogenesis, which are essential for wound closure [6]. However, in chronic wounds, growth factor therapy faces significant obstacles, including the rapid degradation of delivered growth factors, receptor dysfunction, and diminished cellular responsiveness to growth factor signaling within the wound environment [7]. In order to mitigate these challenges and promote effective healing, a multi-faceted approach is essential. Initially, chronic wounds must undergo thorough debridement to remove necrotic tissue and create a wound environment more similar to that of an acute wound [8]. This step is essential as chronic wounds that are stalled in the inflammation phase are often resistant to regenerative therapies [9]. Following debridement, bioactive gels or other formulations containing growth factors and regenerative agents can be applied to accelerate the healing process and promote tissue repair.

One of the current blood-derived therapeutic strategies, Platelet-rich Plasma (PRP), leverages biomolecules from concentrated platelet-rich fractions of autologous blood to enhance tissue regeneration [10]. While PRP has shown promising results in wound healing cases, its efficacy remains variable, and issues, such as the standardization of preparation methods and inconsistent clinical outcomes, continue to impede its widespread adoption [11]. Further strategies to use recombinant growth factor formulations, gene therapy interventions, and stem-cell-based approaches suffer from variable therapeutic efficacy, high costs, and potential adverse effects [12].

In light of these challenges, there is a critical need for innovative approaches that can address the multifaceted nature of wound healing. We have developed a novel method that incorporates hypoxia-induced growth factors from peripheral blood cells (PBCs) into hydrogel matrices, allowing for targeted and sustained factor release [13,14]. Unlike PRP, which primarily contains growth factors from platelets, Hypoxia Preconditioned Serum (HPS) captures a broader range of endogenous signaling molecules and growth factors produced by PBCs under low-oxygen conditions [15,16,17,18]. Moreover, hypoxia is a common physiological condition in various tissues, particularly during wound healing and tissue repair, and is, therefore, a viable strategy to mimic an environment to obtain regenerative signaling molecules [19]. HPS has demonstrated numerous beneficial effects including promoting angiogenesis [17,20,21], lymphangiogenesis [16,18], and the proliferation and migration of fibroblasts [14]. Moreover, in vivo studies have shown enhanced dermal regeneration in mice [22] and positive outcomes in clinical cases of chronic wounds [14,23]. Additionally, studies on osteoblasts and chondrocytes have revealed improved proliferation, migration, and differentiation in vitro [24,25] and the promotion of osteogenesis in a three-dimensional chick bone defect ex vivo [26].

Building on our recent findings of accelerated wound healing in an excisional murine wound model following topical treatment with HPS Hydrogel (HPS-H) [22], this study extends the investigation to a porcine model. In contrast to murine skin, porcine skin is more similar to human skin in thickness, structure, and composition, while dermal regeneration resembles those of humans, rendering porcine models valuable for translational research [27]. However, while our previous research has focused on human HPS, the secretome profile and regenerative effects of porcine HPS remain unexplored. Notably, porcine blood is described as hypercoagulative, and its peripheral immune cell distribution and responses differ from those in humans [28,29]. Although autologous treatment in humans is the primary goal, the use of HPS in xenogeneic or allogeneic context is feasible, given that HPS is cell-free, which minimizes the risk of immunogenicity. Supporting this, studies have demonstrated that allogeneic and xenogeneic PRP releasate, which contains pre-activated, cell-free, platelet-derived growth factors, showed no adverse effects and exhibited superior regenerative outcomes in wound healing as well as in bone and cartilage repair in humans [30,31,32]. Therefore, investigating porcine HPS is particularly relevant, as it can be frozen or lyophilized for on-demand (i.e., off-the-shelf) use in emergency situations, e.g., burns, or for elective treatments in patients with chronic conditions, such as hematological disorders or those on immunosuppressive therapies.

**Figure 1:**
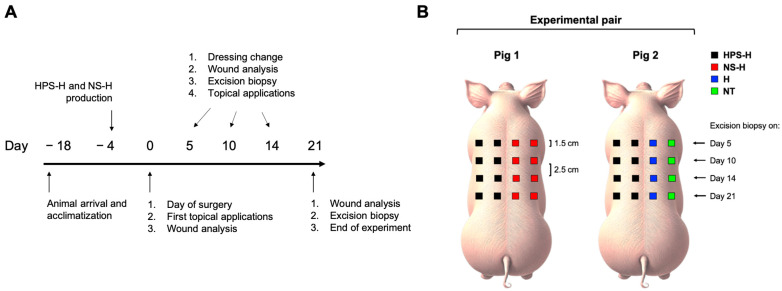
In vivo experimental setup. (**A**) Timeline: At day −18, the animals arrived and were housed in groups for acclimatization. At day −4, Hypoxia Preconditioned Serum Hydrogel (HPS-H) and normal serum hydrogel (NS-H) were produced for each pig and were stored at −20 °C for later use. At day 0, wounding of the pigs was proceeded according to (**B**), and the topical autologous applications (HPS-H and NS-H) and hydrogel only (H) were performed. In the no treatment (NT) group, only saline was applied. Wound analysis included digital photographs and hyperspectral imaging. On days 5, 10, and 14, the dressings were changed, wound analysis was conducted, and, additionally, the respective rows of the indicated days were biopsied for immunohistochemical analysis (see (**B**)). Topical applications were performed for the rest of the wounds. On day 21, final analysis and biopsies were conducted. A total of three independent experiments were conducted, each with two animal pairs.

In this study, we first analyzed the secretome of porcine HPS and evaluated its angiogenic potential using a xenogeneic tube formation assay with human umbilical vein endothelial cells (HUVECs). To confirm a sustained growth factor delivery, we measured the release of regenerative factors from the HPS Hydrogel (HPS-H) over a 5-day period. Autologous HPS-H was applied to porcine wounds and was compared to normal serum hydrogel (NS-H), hydrogel only (H), and no treatment (NT) (Figure 1). Autologous HPS-H was then applied to porcine wounds and compared to normal serum hydrogel (NS-H), hydrogel alone (H), and no treatment (NT) (Figure 1). Wound healing progression was monitored on days 5, 10, 14, and 21 using digital image analysis and hyperspectral imaging wound assessment. Additionally, wedge biopsies were taken at each time point for an immunohistochemical analysis of angiogenesis (CD31), lymphangiogenesis (LYVE-1), and wound contraction (alpha-SMA). With this study, we aim to investigate the therapeutic potential of HPS-H for promoting wound healing in a porcine model.

## 2. Results

### 2.1. Analysis of Growth Factor Concentration in Porcine Hypoxia Preconditioned Serum (HPS) and Its Release from HPS Hydrogel (HPS-H)

**Figure 2:**
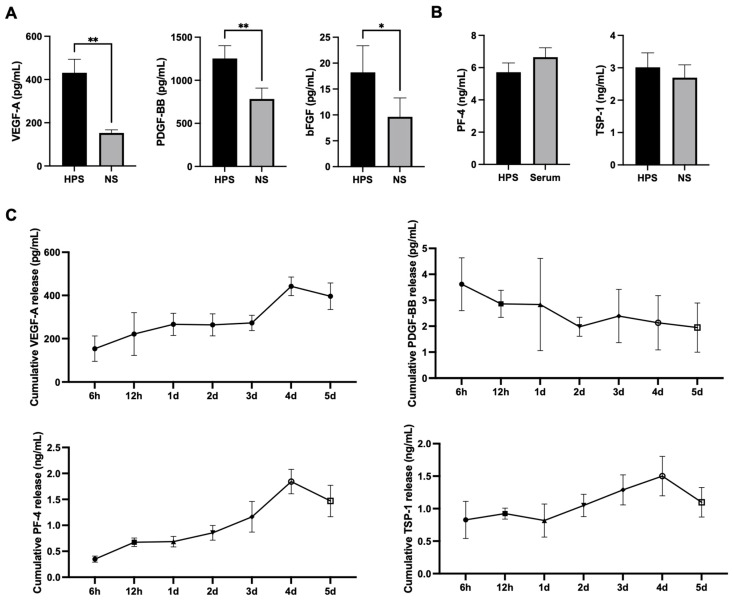
Analysis of growth factor concentration in porcine Hypoxia Preconditioned Serum (HPS) and its release from HPS Hydrogel (HPS-H). (**A**) Quantitative measurements of pro- (VEGF-A, PDGF-BB, and bFGF) and (**B**) anti-angiogenic (PF-4 and TSP-1) growth factors in HPS in comparison to NS. Paired *t*-test. Data presented as mean ± SEM, porcine blood donors: *n* = 6. * *p* < 0.05, ** *p* < 0.01. (**C**) Quantitative analysis of growth factor release in HPS-H over a 5-day period. Data were provided as cumulative concentrations. Data points are means ± SEM. Porcine blood donors: *n* = 4.

In the first step of characterizing growth factors in porcine HPS, we quantitively analyzed the concentration of three pro-angiogenic (VEGF-A, PDGF-BB, and bFGF) and two anti-angiogenic (TSP-1 and PF-4) growth factors in comparison to normal serum (NS) (Figure 2A,B). Here, we found significantly elevated levels of VEGF-A, PDGF-BB, and bFGF in porcine HPS in comparison to porcine NS (431.4 vs. 152.7 pg/mL, *p* = 0.006; 1253.0 vs. 782.5 pg/mL, *p* = 0.009, and 18.22 vs. 9.65 pg/mL, *p* = 0.04, respectively). For the anti-angiogenic growth factor measurements, PF-4 and TSP-1 were not elevated in HPS in comparison to NS. Furthermore, the release measurements from HPS Hydrogel (HPS-H) revealed a consistent release of VEGF-A, PF-4, and TSP-1 from the hydrogel until day 4 and a slight decrease on day 5 (Figure 2C). In contrast, PDGF-BB was observed to release less than 1% of the loading concentration with a slowly decreasing release profile over the 5-day period. Interestingly, bFGF was not measurable in the release media.

### 2.2. The Effect of Porcine HPS on the Tube Formation of HUVECs

**Figure 3:**
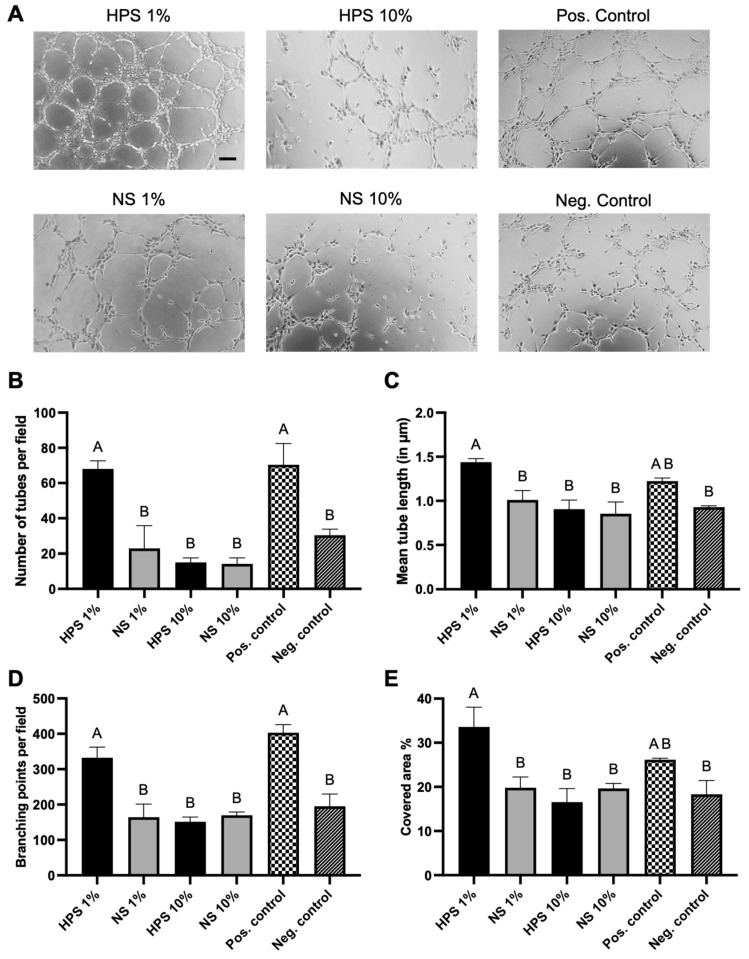
Effect of porcine HPS on the tube formation of HUVECs. (**A**) Representative microscopic photographs of the tube formation assay (6 h) of HUVECs, carried out in the presence of the blood-derived secretomes compared to positive/negative control. Scale bar = 100 μm. (**B**–**E**) Image analysis of the digital photographs depicted in (**A**). One-way ANOVA with Tukey’s multiple comparison test. Data points are means ± SEM. Porcine blood donors: *n* = 3. Capital letters over plots indicate statistical significance of data pairs with different letters. For all pair comparisons, *p* < 0.05.

The angiogenic potential of porcine HPS was evaluated in a xenogeneic tube formation assay using human umbilical vein endothelial cells (HUVECs). HPS was diluted to 1% and 10% with basal media, as these concentrations were found to be most effective in microvessel formation with human HPS in previous studies [14,17]. Here, we found a superior tube-forming capability of higher-diluted HPS-1% in comparison to NS-1%, HPS-10%, NS-10%, and the negative control (basal media), while HPS-1% was comparable to the positive control (growth medium) (Figure 3). In particular, HPS-1% developed 3 times the number of tubes compared to NS-1% (68.0 vs. 22.9, *p* = 0.01) and was also 4.5 times higher than HPS-10% (68.0 vs. 15.0, *p* = 0.005). Similar results were achieved with HPS-1% in mean tube length, which exhibited higher length than NS-1% (1.4 vs. 1.0 μm, *p* = 0.04) and HPS-10% (1.4 vs. 0.9 μm, *p* = 0.008). Correspondingly, HPS-1% gained double the number of branching points than NS-1% (332.2 vs. 164.4, *p* = 0.008) and HPS-10% (332.2 vs. 151.6, *p* = 0.005). With regard to the cell-covered area, the HPS-1%-treated HUVECs displayed the greatest cell-covered percentage, which was significantly greater than NS-1% (33.6 vs. 19.8%, *p* = 0.04) and HPS-10% (33.6 vs. 16.6%, *p* = 0.01). Interestingly, HPS 10% showed no superior angiogenic potential in comparison to NS-10% and was comparable to the negative control.

### 2.3. Wound Closure of Excisional Wounds Treated with Autologous Porcine HPS-H

Having demonstrated the growth factor release of HPS-H and the angiogenic effect of porcine HPS, we administered autologous HPS-H to 1.5 × 1.5 cm^2^ porcine wounds in comparison to normal serum hydrogel (NS-H), hydrogel only (H), and no treatment (NT). Treatments were applied every 4–5 days on day 5, 10, and 14. Wound assessment was performed on each treatment application with a final evaluation on day 21.

**Figure 4:**
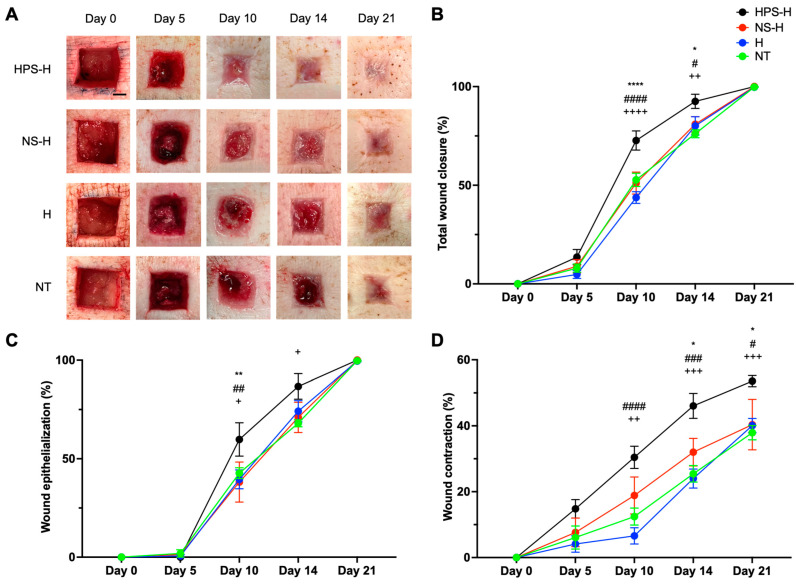
Wound closure of excisional wounds treated with autologous porcine HPS-H in comparison to NS-H, H, and NT. (**A**) Representative standardized photographs of full-thickness wounds in the excisional porcine skin model on postoperative days 0, 5, 10, 14, and 21. Scale bar = 0.5 cm. (**B**) Plot showing total wound closure in % of initial wound surface area (1.5 cm × 1.5 cm). (**C**) Plot showing wound epithelization in % of the contracted wound area. (**D**) Plot showing wound contraction in % of initial wound surface area. (**B**–**D**) Data points represent means ± SEM from three independent animal experiments. Two-way ANOVA with Tukey’s multiple comparisons test. For pair comparisons HPS-H vs. NS-H: * = *p* < 0.05, ** = *p* < 0.01, **** = *p* < 0.0001; HPS-H vs. H: # = *p* < 0.05, ## = *p* < 0.01, ### = *p* < 0.001, #### = *p* < 0.0001; HPS-H vs. NT: + = *p* < 0.05, ++ = *p* < 0.01, +++ = *p* < 0.001, ++++ = *p* < 0.0001.

Using digital photograph analysis, HPS-H-treated wounds exhibited up to a 1.7× increase in total wound closure at day 10 in comparison to NS-H (72.69 vs. 51.08%, *p* < 0.0001), H (72.69 vs. 43.76%, *p* < 0.0001), and NT (72.69 vs. 52.72%, *p* = 0.0002) (Figure 4A,B). On day 14, wounds treated with HPS-H were nearly closed, with the closed wound area significantly greater than wounds treated with NS-H (92.52 vs. 80.91%, *p* = 0.03), H (92.52 vs. 80.10%, *p* = 0.02), and NT (92.52 vs. 76.12%, *p* = 0.001). On day 21, all groups healed with a wound closure higher than 99% (HPS-H and NS-H: 100.0%, H: 99.72%, and NT: 99.80%). 

Further analysis revealed that epithelialization began after day 5 in all groups (Figure 4C). On day 10, HPS-H boosted epithelization considerably up to 36.2% in comparison to NS-H (59.80 vs. 38.16%, *p* = 0.004), H (59.80 vs. 39.54%, *p* = 0.007), and NT (59.80 vs. 42.68%, *p* = 0.03). At day 14, the epithelialization of the HPS-H-treated wounds was less accelerated and only significantly higher than NT (86.71 vs. 68.05%, *p* = 0.01). On day 21, all groups were completely epithelialized (>99%).

Wound contraction, as another aspect of total wound closure, was observed to have increased in all groups by day 5 (Figure 4D). Thereafter, it was significantly enhanced in HPS-H-treated wounds compared to H (30.44 vs. 6.58%, *p* < 0.0001) and NT (31.11 vs. 12.44%, *p* = 0.002) by day 10. On day 14, HPS-H-treated wounds continued to accelerate wound contraction and were significantly more contracted in comparison to NS-H (46.06 vs. 31.99%, *p* = 0.02), H (46.06 vs. 23.99%, *p* = 0.0002), and NT (46.06 vs. 25.43%, *p* = 0.0004). On day 21, NS-H, H, and NT groups demonstrated a similar wound contraction area (37–40%), while HPS-H-treated wounds continued to contract until 53.56% of initial wound size (all *p* < 0.05).

### 2.4. Hyperspectral Analysis of Excisional Wounds Treated with Autologous Porcine HPS-H

**Figure 5:**
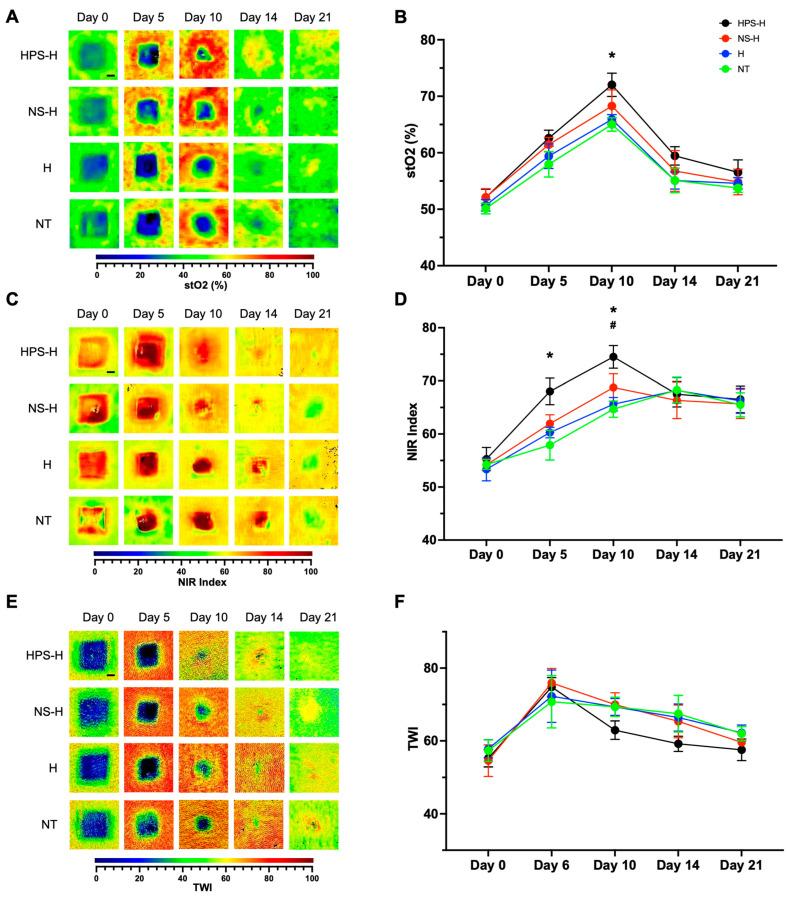
Hyperspectral images of excisional wounds treated with autologous porcine HPS-H in comparison to NS-H, H, and NT. Representative hyperspectral image of (**A**) stO2 (%), (**C**) NIR index, and (**E**) TWI measurements on days 0, 5, 10, 14, and 21. Scale bar = 0.5 cm. The wound edges were analyzed from each day and were plotted in (**B**) for stO2 (%), (**D**) for NIR index, and (**F**) for TWI. (**B**,**D**,**F**) Data points represent means ± SEM from three independent animal experiments. Two-way ANOVA with Tukey’s multiple comparisons test. For pair comparisons HPS-H vs. NT: * = *p* < 0.05; HPS-H vs. H: # = *p* < 0.05.

Further wound analysis was performed using hyperspectral imaging with the TIVITA camera. Parameters included superficial tissue oxygenation (stO2), deep layer perfusion (NIR), and tissue water index (TWI) of the wound edges. StO2 analysis displayed increasing superficial tissue oxygenation in all groups until day 10, whereas HPS-H-treated wounds exhibited a significant 12.8% higher oxygenation than NT (62.03 vs. 55.00%, *p* = 0.04) (Figure 5). After day 10, superficial oxygenation decreased in all groups and reached values similar to those of day 0. NIR measurements exhibited a peak of deep tissue perfusion on day 10 in HPS-H and NS-H-treated wounds and on day 14 in H and NT-treated wounds. Meanwhile, the HPS-H group presented significantly higher deep perfusion than the NT group on day 5 (68.02 vs. 60.56, *p* = 0.04). This trend continued until day 10, whereas the HPS-H group perfused up to 12.9% better than the H group (74.51 vs. 66.61, *p* = 0.03) and the NT group (74.51 vs. 66.00, *p* = 0.02). On day 21, deep perfusion decreased in all groups to a similar level, which was higher than on day 0. For TWI analysis, tissue water exhibited an early peak on day 5 at the wound edges and decreased towards day 21. The HPS-H group demonstrated a lower index after day 10; however, the comparisons with the other groups were not significant.

### 2.5. Immunohistochemical Analysis of Excisional Wounds Treated with Autologous Porcine HPS-H

**Figure 6:**
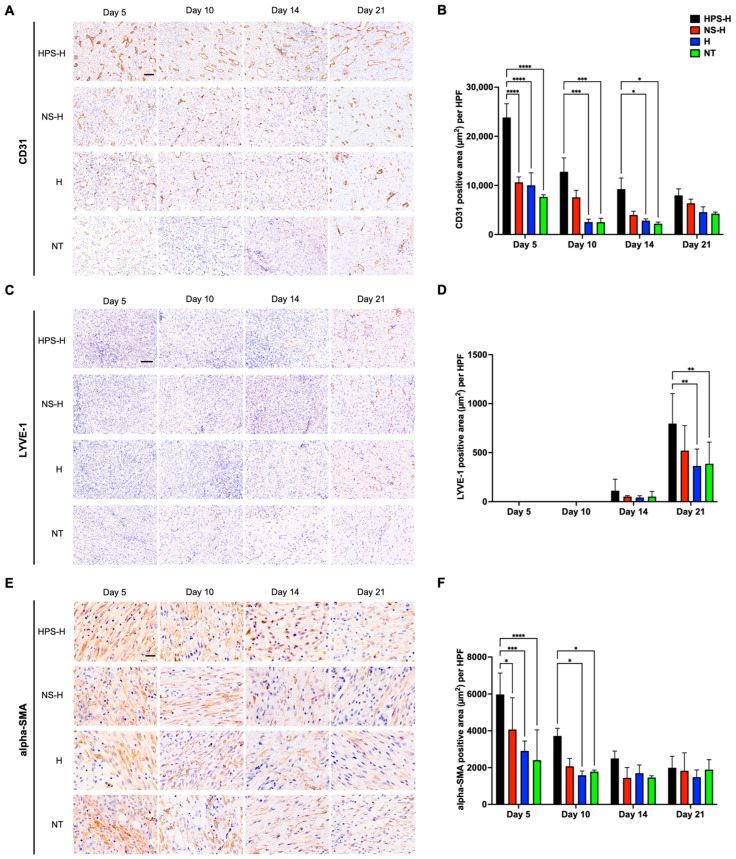
Immunohistochemical diaminobenzidine (DAB)-staining of wounds treated with autologous porcine HPS-H in comparison to NS-H, H, and NT. Representative immunohistochemical staining of (**A**) CD31, (**C**) LYVE-1, and (**E**) alpha-SMA on days 5, 10, 14, and 21. (**A**,**C**) Scale bar = 100 μm and (**E**) scale bar = 33 μm. The area of the DAB-positive staining (μm^2^) was analyzed from each day and were plotted in (**B**) for CD31, (**D**) for LYVE-1, and (**F**) for alpha-SMA. (**B**,**D**,**F**) Data points represent means ± SEM from three independent animal experiments. Two-way ANOVA with Tukey’s multiple comparisons test. * *p* < 0.05, ** *p* < 0.01, *** *p* < 0.001, **** *p* < 0.0001.

We performed immunohistochemical stainings of wound biopsies on days 5, 10, 14, and 21 to gain deeper insight into temporal effects at the histologic level. For angiogenesis analysis, the CD31 staining of day 5 biopsies showed significant vessel formation in the granulation tissue in HPS-H-treated wounds (Figure 6A,B), which was greater than in wounds treated with NS-H (23,839 vs. 10,621 μm^2^, *p* < 0.0001), H (23,839 vs. 10,009 μm^2^, *p* < 0.0001), and NT (23,839 vs. 7666 μm^2^, *p* < 0.0001). Notably, vessel density decreased on day 10 in all groups, but larger and more mature vessels were formed in the HPS-H-treated wounds which showed a greater CD31 staining area than in the H (12,778 vs. 2536 μm^2^, *p* = 0.0002) and NT groups (12,778 vs. 2498 μm^2^, *p* = 0.0002). This trend was continued on day 14, where HPS-H-treated wounds exhibited a greater CD31 stained area than H (9236 vs. 2820 μm^2^, *p* = 0.03) and NT groups (9236 vs. 2498 μm^2^, *p* = 0.01). On day 21, the vessels in the NS-H, H, and NT groups displayed increased CD31 staining compared to day 10. However, their CD31 staining area was relatively lower than in the HPS-H-treated wounds, but this was of no significance.

To analyze lymphangiogenesis, the tissues were stained for LYVE-1. Interestingly, no lymphatic cell invasion was detected in the granulation tissue until day 10 (Figure 6C,D). On day 14, lymphatic vessels began to form in the granulation tissue in all of the groups. On day 21, HPS-H-treated wounds exhibited a significantly more LYVE-1 staining area than in the H (796.5 vs. 364.4 μm^2^, *p* = 0.001) and NT groups (796.5 vs. 386.1 μm^2^, *p* = 0.002).

Alpha-SMA staining was performed to detect myofibroblasts and thus analyze the degree of wound contraction at the histological level (Figure 6E,F). On day 5, a strong detection of alpha-SMA in fibroblasts was observed in all groups, with higher levels in HPS-treated wounds compared to NS-H (5974 vs. 4069 μm^2^, *p* = 0.03), H (5974 vs. 2905 μm^2^, *p* = 0.0003), and NT (5974 vs. 2404 μm^2^, *p* < 0.0001). The detection of alpha-SMA decreased on day 10, with the HPS-H-treated wounds achieving higher levels than H (3723 vs. 1586 μm^2^, *p* = 0.01) and NT (3723 vs. 1779 μm^2^, *p* = 0.03). On days 14 and 21, alpha-SMA was detected equally in all groups.

## 3. Discussion

This study investigates the therapeutic potential of autologous Hypoxia Preconditioned Serum (HPS) integrated into a hydrogel (HPS-H) for promoting wound healing utilizing a porcine model. The results demonstrate that HPS-H significantly accelerates wound closure, particularly in the early stages of healing compared to normal serum hydrogel (NS-H), hydrogel only (H), and no treatment (NT). The enhanced wound healing effects of HPS-H treatment can be attributed to its multifaceted influence on key aspects of the wound healing process, including enhanced angiogenesis, lymphangiogenesis, and wound contraction, as evidenced by the immunohistochemical analyses. The findings highlight the potential of HPS-H as a novel therapeutic strategy for wound care, leveraging the unique regenerative properties of hypoxia-induced growth factors.

The study revealed that porcine HPS contained significantly higher levels of pro-angiogenic and regenerative factors, particularly VEGF-A, PDGF-BB, and bFGF, in comparison to normal serum (NS). This effect is consistent with findings in human HPS [16,17,25], suggesting a replicable mechanism of hypoxic preconditioning of PBCs in a porcine setting. Interestingly, the absolute concentrations of growth factors in both porcine HPS and NS were 3–10 times lower than in the corresponding human-blood-derived compositions [13,14,16,17,25]. In contrast, the anti-angiogenic factors TSP-1 and PF-4 were not elevated in porcine HPS, as opposed to their upregulation in human HPS [16]. Both TSP-1 and PF-4 are protein factors that are stored in the alpha granules of platelets and recognized for their anti-angiogenic properties by inhibiting endothelial cell proliferation and migration [33,34]. Comparative studies on TSP-1 and PF-4 between humans and pigs are limited; however, the observed differences in their regulation under hypoxic conditions may be attributed to species-specific differences in platelet biology. Porcine blood is known to be hypercoagulable in comparison to human blood, likely due to a higher platelet count and enhanced platelet aggregation, potentially driven by elevated levels of von-Willebrand factor [35,36]. Furthermore, as porcine wound healing tends to be faster than in humans [37], the reduced levels of anti-angiogenic factors following hypoxic preconditioning of PBCs may support more robust blood vessel formation, leading to optimized wound healing. This may reflect an evolutionary adaptation in pigs, where rapid wound healing may be advantageous due to different environmental pressures or physiological demands [37]. Nonetheless, the mechanisms of hypoxia-induced signaling and platelet factor release in pigs require further exploration.

The use of alginate hydrogel as a delivery matrix for HPS growth factors presents several advantages for clinical wound healing applications. Alginate, a naturally derived polysaccharide from brown algae or bacteria, is well recognized for its biocompatibility, biodegradability, and ability to form hydrogels, rendering it an ideal candidate for the sustained release of bioactive molecules [38,39]. The promotion of wound healing through the effects of alginate hydrogel alone has been confirmed by several studies: These effects are dependent on wound cellular debridement by macrophage activation, the dissolution of necrotic/fibrotic tissue, and wound rehydration [40,41]. The continuous release of growth factors from HPS-H over a five-day period further supports the feasibility of this approach for therapeutic wound dressings. The gradual release of VEGF-A, in particular, aligns with the need for prolonged angiogenic stimulation during the wound healing process. Notably, PDGF-BB exhibited a slower release profile, which may be attributed to its active binding to the hydrogel, a pattern also observed in HPS-fibrin formulations [26]. However, the release of bFGF from the hydrogel was not measurable, possibly due to the very low levels detected in porcine HPS and NS. Overall, our findings are consistent with previous studies, such as those using PRP-loaded alginate hydrogel, which demonstrated a sustained release of growth factors for up to 3 days [42], as well as other studies incorporating individual growth factors into alginate hydrogels that allowed release over 7–10 days [43,44].

The potential of porcine HPS to promote xenogeneic angiogenesis was confirmed using a tube formation assay with HUVECs, where HPS at 1% concentration significantly outperformed normal serum in promoting tube formation. Interestingly, HPS-1% showed superior performance compared to HPS-10%, suggesting a dose-dependent effect where lower concentrations may be more effective. This may be due to a more optimal balance between pro- and anti-angiogenic factors and/or reaching the ideal dose of pro-angiogenic activity. Although the anti-angiogenic factors TSP-1 and PF-4 were not increased in HPS after hypoxic incubation, the concept of “angiogenic disinhibition” by dilution of their inhibitory effect remains plausible. This is supported by the observation that HPS-10% performed similarly to NS-10% and the negative control. These findings are consistent with our previous results in human blood, where progressively stronger angiogenic responses were seen as HPS was diluted to 1%, with diminished effects at higher dilutions [14,17]. This contrasts with PRP studies, where the greatest angiogenesis was observed at 25-50% dilution [45]. Furthermore, the comparable performance of HPS-1% to the positive control (recombinant VEGF-A) in promoting tube formation highlights the potential of HPS as a potent angiogenic therapy, particularly in a xenogeneic setting.

In the porcine wound model, HPS-H treatment significantly improved wound closure rates compared to NS-H, H, and NT. By day 10, HPS-H-treated wounds exhibited considerable wound closure, evidenced by higher wound contraction and epithelialization, which are critical parameters in assessing wound healing efficacy. This enhanced healing effect can be attributed to the sustained release of growth factors from HPS-H, creating an optimal environment for cellular activities essential for wound repair. The ability of HPS-H to promote wound closure more effectively than NS-H highlights the added value of hypoxia-preconditioned factors in boosting therapeutic efficacy. These findings align with previous research on murine wound healing [22] but extend them by demonstrating efficacy in a large animal model that more closely resembles human skin. In comparison, a study of PRP-loaded hydrogel in minipigs reported similar wound healing dynamics for larger wounds of twice the size (3 × 3 cm^2^), with faster closure rates after day 11 [46]. Conversely, another study involving PRP and platelet-rich fibrin (PRF) applications in minipigs with 3 × 3 cm^2^ wounds did not accelerate wound healing [47]. In another minipig model using 2 × 2 cm^2^ exposed-bone skull wounds, PRP incorporated into a dermal scaffold achieved significantly higher wound healing rates by day 10 [48]. In the context of human wound healing, the efficacy of HPS-H was supported by a retrospective clinical study, involving six surgical cases, two of which featured extensive full-thickness dermal wounds caused by postoperative skin necrosis [23]. These results suggest that HPS-H treatment effectively targets key cellular regenerative processes, accelerating wound healing. 

To achieve a more comprehensive wound assessment in this study, we employed the TIVITA hyperspectral camera, a medical device that analyzes superficial oxygenation levels of the blood (StO2), deeper blood perfusion using the near-infrared index (NIR), and water content in the tissues using the tissue water index (TWI). The analysis of StO2 and NIR revealed increasing tissue oxygenation and deep perfusion across all groups until day 10, with HPS-H-treated wounds displaying significantly higher levels. Interestingly, these findings correlate with the significant acceleration of epithelialization and wound contraction observed on day 10. This suggests, that the improved tissue perfusion and enhanced oxygen delivery, resulting from the angiogenic effect of HPS-H treatment, may facilitate the transport of essential nutrients and immune cells to the wound site, thereby promoting a conducive environment for wound healing. The bell-shaped pattern of oxygenation and perfusion measurements, peaking between days 10 and 14, aligns with existing literature utilizing laser Doppler perfusion in porcine wound models [49,50]. Interestingly, a study on PRP hydrogel treatment in 3 × 3 cm^2^ porcine wounds indicated an earlier significant increase in wound perfusion, starting at day 3 and continuing until day 14 [46]. To our knowledge, only a limited number of cases in the literature have reported the use of TIVITA for the analysis of wound healing phases, particularly in the context of blood-derived treatments. One study evaluating wound healing in a full-thickness murine wound model following argon-plasma treatment demonstrated a significant increase in StO2 immediately after the treatment, with an overall pre-treatment increase in StO2 on day 12, while the NIR and TWI remained largely unchanged during the 15-day observation period [51]. This study was replicated in both male and female mice showing only a direct increase in StO2 following argon-plasma treatments [52]. Additionally, a systematic review reinforces the importance of advanced imaging techniques like hyperspectral imaging in wound care, suggesting that such methodologies can enhance the understanding of wound healing outcomes and the effectiveness of various therapeutic interventions [53]. The findings from these studies highlight the potential of TIVITA not only as a diagnostic tool but also as a valuable approach for optimizing treatment strategies in wound management, especially when used alongside novel therapies such as HPS treatments.

In the immunohistochemical analysis, we examined the staining of CD31, LYVE-1, and alpha-SMA at days 5, 10, 14, and 21. CD31 staining revealed a significant increase in (micro-)vessel formation within the granulation tissue of wounds treated with HPS-H on day 5, exceeding levels observed in wounds treated with NS-H, H, and NT. Subsequently, CD31 staining decreased in all groups, with HPS-H remaining significantly higher through day 14, while exhibiting larger vessel lumens compared to the other groups. These findings align with the improved StO2 and NIR measurements and improved wound healing in HPS-H-treated wounds. By day 21, the CD31 expression of NS-H, H, and NT groups gradually increased to levels comparable to those in the HPS-H group. This observation is consistent with the murine wound healing studies, where CD31 expression in healed wounds on day 14 was similar between HPS-H and control groups [22]. Another study examining full-thickness wounds (2 × 2 cm^2^) in a pig model, healing by secondary intention also noted the highest vessel density early after wounding (day 6), followed by significant decrease due to vessel maturation [54]. LYVE-1 immunostaining revealed no lymphatic cell invasion in the granulation tissue until day 10. Lymphatic vessel formation began to emerge in all groups by day 14, with significantly greater LYVE-1 staining in HPS-H-treated wounds than in the H and NT groups by day 21. Although angiogenesis and lymphangiogenesis are related processes, they are distinct and initiate at different times, responding to different biochemical signals [55,56]. During wound healing, the repair of the lymphatic network occurs later than angiogenesis and is driven by specific factors, such as VEGF-C [57], which was detected at high levels in human HPS [16]. Our previous mouse model study similarly demonstrated higher LYVE-1 expression in HPS-H-treated wounds at day 14 [22]. Another study also reported a late upregulation of LYVE-1 in murine wounds at day 14 in VEGF-C treated mice [58]. Alpha-SMA staining revealed a significant increase in myofibroblasts within the granulation tissue of wounds treated with HPS-H on day 5 and day 10, surpassing levels seen in wounds treated with NS-H, H, and NT. Following this peak, alpha-SMA expression decreased by days 14 and 21, reaching similar levels in all groups. This trend aligns with the digital photograph analysis, which indicated accelerated wound contraction until day 14 in HPS-H-treated wounds. A study of porcine skin wounds similarly demonstrated that increased alpha-SMA was associated with wound contraction, suggesting an active role for myofibroblasts in the healing process [54]. Moreover, the same study revealed similarly that alpha-SMA expression peaked between day 6 and 18 post-wounding, corresponding to the transition from the inflammatory to the proliferative wound phase [54]. The early upregulation of alpha-SMA was also observed in murine wounds, both in the treatment groups (povidone-iodine application, PPAR gamma deletion) and control groups, with the treatment group exhibiting significantly higher alpha-SMA expression [59,60].

While this study provides valuable insight into the therapeutic potential of HPS-H, it is not without limitations. The small sample size and the absence of a chronic wound healing model (e.g., infected wound model and diabetic wound model) may restrict the generalizability of the findings. Our clinical experience with HPS-H in diabetic wounds and wound healing disorders (e.g., postoperative skin necrosis and wound dehiscence) is based on the proper surgical debridement of the chronic wound to convert a chronic wound into an acute wound. Following acute wounding, HPS-H has been shown in human preclinical studies to significantly accelerate the healing of chronic wounds [14,23]. Future studies should also include head-to-head comparisons with other regenerative therapies to better understand the benefits of HPS-H. In addition, while the study focused on short-term aspects of wound healing, it lacked a thorough examination of scar formation, which is critical to the overall quality of wound healing [61]. Finally, the results of this research need to be tested in human clinical trials.

## 4. Conclusions

This study provides evidence supporting the use of Hypoxia Preconditioned Serum integrated into a hydrogel matrix (HPS-H) as a potent therapeutic approach for enhancing wound healing. The sustained release of key growth factors, superior angiogenic potential, and the significant improvements in wound closure observed in the porcine model underscore the potential of HPS-H as an innovative treatment for tissue repair. The ability to produce HPS from autologous blood, combined with the ease of integrating it into a hydrogel for sustained release, makes it a practical option for widespread clinical use.

## 5. Materials and Methods

### 5.1. Ethical Approval

All in vivo experiments were performed under the guidance of veterinarians of the Center for Preclinical Research (ZPF) of the Technical University of Munich, Germany, and were approved by the Ethical Committee for Animal Experiments of the Government of Upper Bavaria (ROB-55.2-2532.Vet_02-17-214; date of addendum approval: 16 February 2023).

### 5.2. Production of Porcine Hypoxia Preconditioned Serum (HPS) and HPS Hydrogel (HPS-H)

HPS was prepared according to our previous protocols [14]. Briefly, a 30 mL polypropylene syringe (Omnifix, B. Braun AG, Melsungen, Germany) was used to collect 20 mL of peripheral venous blood under sterile conditions via a central venous catheter, which was inserted at day −4 (Figure 1). Subsequently, 5 mL of air was drawn into the syringe through a 0.2 μm filter (Sterifix, B. Braun AG, Melsungen, Germany) under laminar flow conditions. A closed chamber was then created by sealing the syringe. Syringes were incubated upright (37 °C, 5% CO_2_) for 4 days to create pericellular hypoxia <1% O_2_, as previously described [13,14,19]. After incubation, the blood was separated into two layers (serum and clot). The upper layer consisted of HPS. This layer was filtered into a new syringe to remove cell particles and debris, resulting in a cell-free product. HPS-H was prepared by mixing 40% HPS and 60% commercially available sodium-alginate hydrogel (Nu-Gel, KCI GmbH, Wiesbaden, Germany). HPS-H was aliquoted and stored at −20 °C prior to experimental testing.

### 5.3. Production of Porcine Normal Serum (NS) and NS Hydrogel (NS-H)

Corresponding normal serum (NS) was prepared from the same donors as for HPS. Briefly, 20 mL of peripheral venous blood was collected into a 30 mL polypropylene syringe (Omnifix, B. Braun AG, Melsungen, Germany) from the central venous catheter under sterile conditions. The blood was left upright at room temperature (21 °C) for 4 h to allow sedimentation and clotting of peripheral blood cells. The serum was then filtered through a 0.2 μm filter (Sterifix, B. Braun AG, Melsungen, Germany) under laminar flow conditions. NS-H was prepared by mixing 40% NS with 60% commercially available sodium-alginate hydrogel (Nu-Gel, KCI GmbH, Wiesbaden, Germany). NS-H was aliquoted and stored at −20 °C prior to experimental testing.

### 5.4. Quantification of Growth Factors in Porcine HPS and NS

Enzyme-linked immunosorbent assay (ELISA) was performed for the quantitative measurements of the following growth factors in porcine HPS and NS: vascular endothelial growth factor-A (VEGF-A), platelet-derived growth factor-BB (PDGF-BB), basic fibroblast growth factor (bFGF), platelet-factor-4 (PF-4), and thrombospondin-1 (TSP-1). The corresponding ELISA kits were utilized according to the manufacturer’s protocol (ABIN6971118 for VEGF-A, ABIN6966763 for bFGF, ABIN779967 for TSP-1, ABIN779558 for PF-4, antibodies-online Inc., Pottstown, PA, USA, and ELP-PDGFB-1-RB for PDGF-BB, RayBiotech, Peachtree Corners, GA, USA). Readout was performed by optical density measurement at a 450 nm wavelength using Mithras LB 940 Multimode Microplate Reader (Berthold Technologies GmbH & Co. KG, Bad Wildbad, Germany). All conditions were tested in duplicates per blood donor, and a total of six porcine donors were taken for evaluation.

### 5.5. Quantification of HPS-H Growth Factor Release

The release of growth factors from the HPS-H was evaluated over a period of 5 days. For the assay, HPS-H was prepared as described in Section 5.2, and 1 mL was transferred into a 5 mL tube (Eppendorf, Hamburg, Germany), to which 1 mL PBS was added. The tubes were incubated at room temperature on a rocking plate. Supernatants were collected after 6 h and 12 h and 1, 2, 3, 4, and 5 days. The samples were stored at −80 °C until the ELISAs were performed. The growth factors investigated were vascular endothelial growth factor-A (VEGF-A), platelet-derived growth factor BB (PDGF-BB), and basic fibroblast growth factor (bFGF). ELISAs were performed according to the manufacturing protocol (ABIN6971118 for VEGF-A, ABIN6966763 for bFGF, ABIN779967 for TSP-1, ABIN779558 for PF-4, antibodies-online Inc., Pottstown, PA, USA, and ELP-PDGFB-1-RB for PDGF-BB, RayBiotech, Peachtree Corners, GA, USA). Readout was performed by optical density measurement at a 450 nm wavelength using the Mithras LB 940 Multimode Microplate Reader (Berthold Technologies GmbH & Co. KG, Bad Wildbad, Germany). All conditions were tested in duplicates per blood donor, and a total of four porcine donors were taken for evaluation.

### 5.6. Tube Formation Assay

The angiogenic potential of porcine-derived blood products was evaluated in a xenogeneic in vitro assay by assessing their capacity to induce tube formation in human umbilical vein endothelial cells (HUVECs). In brief, HUVECs were subjected to overnight starvation in AIM V basal medium (12055091, Thermo Fisher, Waltham, MA, USA). On the following day, the cells were resuspended in sample media (1%, 10% of HPS/NS diluted with AIM V basal media), Endothelial Cell Growth Medium (C-22010, PromoCell, Heidelberg, Germany) as positive control, and AIM V basal medium as negative control. Subsequently, 50 μL of the cell-medium mixture was seeded onto μ-Slides (81506, Ibidi GmbH, Martinsried, Germany) previously coated with 10 μL of reduced growth factor Matrigel (356231, Corning Inc., Corning, NY, USA) at a density of 40,000 cells per cm^2^. Following a six-hour incubation period at 37 °C and 5% CO₂, images from each well were captured using an inverted phase-contrast microscope (Axio Vert.A1, Carl Zeiss, Jena, Germany). The extent of capillary-like network formation was evaluated using the image analysis tool “IKOSA AI” (KOLAIDO, Altenrhein, Switzerland). This tool quantified the number and total length of tubes, the number of branching points (defined as the point of intersection of two or more tubules), and the cell-covered area from four high-power fields (HPFs) captured per well. Subsequently, the mean value of the HPFs was calculated for each well. All conditions were tested in triplicates per blood donor, and a total of three porcine donors were taken for evaluation.

### 5.7. Animals

Six 52-week-old adult minipigs (Aachen minipigs) were obtained from Heinrichs Tierzucht GmbH (Heinsberg, Germany). These minipigs are especially suited for scientific studies of wound healing, as their growth-related influences on wound healing in adulthood are minimal. The minipigs were housed under controlled environmental conditions, including temperature, humidity, and light, with a 12 h light/dark schedule in the AAALAC (Association for Assessment and Accreditation of Laboratory Animal Care)-accredited animal facility of the Center for Preclinical Research of the Technical University of Munich, Germany. The animals were fed a standard minipig diet in pellet form twice a day and had access to fresh tap water via automatic nipple drinkers at all times. Prior to the commencement of the study, the pigs were housed for a minimum of 14 days to allow for acclimation to the novel environment and personnel. The pigs’ health was monitored on a daily basis using a standardized scoring system involving defined parameters, e.g., general condition and behavior, food and water intake, and weight measurement. Following surgical procedures, the pigs were housed individually to prevent any manipulation of the wound dressings.

### 5.8. Full-Thickness Wound Procedure

The methodology for creating full-thickness wounds was adapted from a minipig wound model described by O’Brien [62]. The treatment/control groups were positioned on either side of the spine, with the blood circulation separated, thus enabling two treatment and two control groups to be compared. Two minipigs were paired for an experimental run, with one treatment group (HPS-H) and three control groups (NS-H, H, and NT). Group 1 consisted of HPS-H and NS-H, while Group 2 consisted of HPS-H and H/NT on both sides of the spine (Figure 1B). H and NT were allocated to the same side in Group 2, as they do not release biomolecules that could potentially interfere with one another. Each side of the spine comprised eight wounds in four rows, with each wound measuring 1.5 × 1.5 cm. The pigs were premedicated with azaperone (2 mg/kg), ketamine (15 mg/kg), and atropine (0.1 mg/kg) intravenously via the central venous catheter. Propofol 1% was then injected as a bolus (4–8 mg/kg), depending on effect. Intubation was then performed. A continuous infusion of propofol 2% was then administered to maintain anesthesia. As part of the induction of anesthesia, metamizole (40–50 mg/kg, i.v.) was administered, followed by fentanyl (0.001–0.01 mg/kg every 20–30 min, i.v.). As infection prophylaxis, the animals were administered cefuroxime before the first skin incision (750 mg/animal, i.v.). For the surgical planning, the wounds were marked with a preformed template. The distance between two wounds was 2.5 cm. Following the removal of hair and the application of octenisept solution (Schülke & Mayr GmbH, Norderstedt, Germany) for disinfection, the dermis and subcutis were excised with a scalpel to expose the underlying fascia. The depth of the incision was approximately 1 cm. Aliquoted HPS-H and NS-H were thawed at room temperature. Subsequently, all treatments and controls were administered locally. All wounds were separated from one another with the use of strips and are then covered with Tegaderm (3M, Saint Paul, MN, USA). Additional fixation was achieved through the use of staples in the region of the strips. Subsequently, a foam dressing (Granufoam, 3M, Saint Paul, MN, USA) was applied over the aforementioned area and secured with staples. An absorbent compress was then applied for padding purposes, followed by the application of a custom-made pig vest. The purpose of the vest was to provide protection and stabilization for the underlying dressing layers. Postoperatively, a fentanyl patch (3–4 µg/kg/h, 3 days duration of action) was applied, and buprenorphine (0.005–0.1 mg/kg) was administered intravenously every 4–8 h for 12 h.

### 5.9. Wound Analysis

Wound analysis was conducted under general anesthesia on days 5, 10, 14, and 21 during dressing changes and prior to excisional biopsy for immunohistochemistry. The same anesthetic protocol and pain management was used as in Section 5.8. Digital photographs and hyperspectral images were captured with the TIVITA camera (Diaspective Vision, Am Salzhaff, Germany) at a fixed distance of 50 cm, as indicated by the camera’s integrated distance locator. Each hyperspectral image was acquired in accordance with standardized conditions, specifically in a darkroom with the same exposure times. The wound area parameters, namely, total wound area, epithelialization, and wound contracture, were analyzed through the tracing of the inner margin, which represents the extent of total wound healing, and the outer margin, which represents the extent of contraction. The areas were then calculated using the ImageJ software (version 1.53, NIH, Bethesda, MD, USA). The percentage of total wound closure was calculated by dividing the initial wound area minus the inner margin wound area by the initial wound area. The percentage of wound epithelialization was calculated by subtracting the inner margin wound area from the outer margin wound area and dividing the result by the outer margin wound area. The percentage of wound contraction was calculated by subtracting the outer margin wound area from the initial wound area and dividing the result by the initial wound area. The parameters of the hyperspectral camera images were measured as the mean of the four wound edges of each wound. The mean value of all wounds from each treatment group was calculated for each experimental run.

### 5.10. Immunohistochemistry

Subsequent to the wound analysis delineated in Section 5.9, the corresponding row of wounds was excised via surgical intervention under general anesthesia (Figure 1B), with the excised area measuring 2 × 2 cm^2^. The resulting wounds were then closed with interrupted sutures using Prolene suture size 1 (Ethicon, Raritan, NJ, USA). The square-shaped tissue samples were bisected into identical halves and immediately fixed in 4% paraformaldehyde at 4 °C for three days. The samples were then dehydrated in a series of ethanol solutions, embedded in paraffin, and serially cut into sections of 4 μm thickness. The sections were examined on a coated slide glass and were first stained with hematoxylin and eosin (H&E) for gross investigation. Immunohistochemical staining for the endothelial cell marker CD31 (ab28364, Abcam, Cambridge, UK), the lymph-endothelial cell marker LYVE-1 (ab33682, Abcam, Cambridge, UK), and the wound contraction marker alpha-SMA (ab5694, Abcam, Cambridge, UK) were conducted on the fully automated Bond-Max system (Leica Biosystems, Nussloch, Germany). The immunohistochemical antibodies were subsequently subjected to the two-step peroxidase technique, with the staining process conducted using diaminobenzidine (DAB) chromogen. The slides were digitally scanned using a Leica Aperio microscope (Leica Biosystems, Nussloch, Germany). The immunohistochemistry slides were analyzed in accordance with the methodology described by Mezei et al. [63]. By employing the hue/saturation/brightness color filtering tool in ImageJ, it was possible to quantify the area of tissue marked by DAB staining. In summary, the following settings were employed (numbers indicate the minimum and maximum values, while letters in brackets indicate the filter type): P (pass) and S (stop) indicate the appropriate settings for the process. The hue was set to 44/255 (S), the saturation to 37/255 (P), and the brightness to 0/255 (P). A high-powered field (HPF) was selected at 3000 × 3000 pixels for CD31 and LYVE-1 and 1000 × 1000 pixels for alpha-SMA. Each tissue sample was subjected to analysis using four randomly selected high-powered fields (HPFs). Subsequently, the areas marked by DAB staining were calculated in pixels and converted into μm² metrics.

### 5.11. Statistical Analysis

GraphPad Prism version 10 software (GraphPad, Boston, MA, USA) was used for statistical analysis. Data sets were analyzed by paired *t*-test if two comparison groups were available. In order to test the suitability of the ANOVA model, a Shapiro–Wilk test was conducted to assess the normality of the data, while a Brown–Forsythe test was employed to evaluate the equality of variances. One-way analysis of variance (ANOVA) with subsequent comparisons using Tukey’s post hoc analysis was used when there were more than two groups and one independent variable. If two independent variables were present, a two-way ANOVA with subsequent comparisons using Tukey’s post hoc analysis was performed. All values are expressed as means ± standard error of the mean (SEM). A value of *p* < 0.05 is considered statistically significant.

## 6. Patents

Device-based methods for localized delivery of cell-free carriers with stress-induced cellular factors. (AU2013214187 (B2); 9 February 2017: Schilling Arndt, Hadjipanayi Ektoras, Machens Hans-Günther).

## Data Availability

The data that support the findings of this study are available from the corresponding author upon request.
